# Schistosomiasis and soil-transmitted helminthiasis prevalence and associated factors among school children in the Hawela Tula sub-city, Ethiopia: a cross-sectional study

**DOI:** 10.3389/fepid.2025.1514964

**Published:** 2025-11-25

**Authors:** Addisalem Wube, Tsegaye Alemu, Tarekegn Solomon

**Affiliations:** 1 School of Public Health, Yanet-Liyana College of Health Sciences, Hawassa, Ethiopia; 2 School of Public Health, Hawassa University, Hawassa, Sidama, Ethiopia

**Keywords:** factors, prevalence, primary school children, schistosoma, soil transmitted helminthiasis, Hawela Tula sub city

## Abstract

**Background:**

Intestinal helminthiasis is a neglected tropical disease that affects more than 1.5 billion people worldwide, and school-aged children are particularly vulnerable. However, there is *limited local evidence in Hawela Tula sub city.*

**Objectives:**

To identify prevalence and factors associated with Schistosomiasis and Soil-Transmitted Helminthiasis among schoolchildren at the Primary School in Hawassa, Hawela Tula sub city, Sidama Region, Ethiopia.

**Methods:**

A school-based cross-sectional study was conducted from October 1 to November 30, 2023. The study used a multistage random sampling technique to select 740 participants. The data were collected via face-to-face interviews using the Kobo collection tool with a smartphone. Stool samples were collected from students and then processed and examined using direct wet mount microscopy and formol-ether concentration techniques. The data were checked for completeness and consistency and then coded and entered into SPSS Version.27, for analysis. Descriptive statistics were computed to describe the data. Bivariate and multivariate logistic regression models were used to assess factors associated with intestinal helminthic infections.

**Results:**

A total of 726 study respondents were included, yielding in a response rate of 98.1%. The mean (±SD) age of the study participants was 8.8 (±2.1) years. The overall prevalence of intestinal helminthic infections among school children was 39.5% (95% CI: 36.0–43.1). The major identified intestinal helminthic parasite species were *Ascaris lumbricoides* (43.9%), *Schistosoma mansoni* (26.1%), hookworm species (8.7%), multiple infections (8.7%), and *Trichuris trichuria* (8.4%). After adjusting for covariates, being in lower grade (1–2) (AOR = 1.53, 95% CI: 1.12–2.10), having a mother with no formal education (AOR = 1.50, 95% CI: 1.05–2.15), having untrimmed fingernails and not clean (AOR = 1.84, 95% CI: 1.12–3.01), not washing hands before meals (AOR = 1.90, 95% CI: 1.35–2.68) and eating unwashed vegetables (AOR = 1.58, 95% CI: 1.11–2.24) were significantly associated with intestinal helminthic infection.

**Conclusion:**

This study revealed that four out of ten schoolchildren were infected with intestinal helminthiasis. Children in lower grades born to mothers with no formal education, with untrimmed fingernails and poor hygiene, not practising hand washing before meals, and consuming raw meat and unwashed vegetables were found to be more susceptible to intestinal helminthic infection. To address soil transmitted helminthiasis and Schistosomasis diseases effectively, local governments, regional health bureaus, and development partners should prioritize targeted interventions and implement innovative strategies to reduce their burden. At the community level, schools and families can play a critical role by reinforcing proper hygiene and sanitation practices among children to tackle diseases.

## 1 Introduction

Intestinal helminthiasis infections are a major concern for low- and middle- income countries and causing significant proportion of morbidity and mortality. Most infectious diseases caused by members of the intestinal parasites (protozoan and helminthes) have been considered as Neglected Tropical Diseases (NTDs) (1, 2). Soil-transmitted helminths (STHs) and schistosomiasis are among the NTDs significantly affect millions of people worldwide, particularly school-aged children in low- and middle-income countries located in tropical and subtropical regions (3–6). STHs are caused by various nematodes, such as *Ascaris lumbricoides*, *Trichuris trichiura*, and hookworms (*Necator americanus* and *Ancylostoma duodenale*) (3).

Globally, an estimated 4.5 billion people are at risk of intestinal helminth infections, approximately 1.5 billion people are infected with STHs. Approximately 300 million people suffer from severe morbidity attributed to intestinal helminth infections, resulting in 10,000–135,000 deaths each year (7). The highest burden of the infection occurs in sub-Saharan Africa (8), China and East Asia (4). An estimated 270 million pre-school children and over 600 million school-aged children live in areas where these parasites are widely transmitted (9). The estimated number of people known to be infected globally is 807–1,121 million with *Ascaris*, 604–795 million with whip-worm, 576–740 million with hookworm and 240 million with *Schistosomiasis* (4). In sub-Saharan Africa, approximately 198, 192, 173, and 162 million people are infected with *hookworms, Schistosomiasis species, A. Lumbricoides and T. Trichiura,* respectively (10).

STHs primarily affect populations with inadequate sanitation and hygiene practices (11). Effective WASH programs promote behaviors such as proper sanitation practices, hand washing with soap at critical times and using safe drinking water. These behaviors disrupt the life cycle of STHs, preventing the spread of infection and contributing to long-term control (12). Pre-school and school aged children are the most vulnerable age group (13, 14). This is because their typical hand-mouth activity, water contamination and their immature immune system. Moreover, their behavioral activities are also associated with the high prevalence of STHs compared to adults (8, 15). Adults are not exempt from getting infected. Different previous studies from Ethiopia and Ecuador showed high prevalence of STH infection among the adult population ranging from 31.2% to 65% (16, 17).

Intestinal helminth infections causing a wide range of nutritional, physical, and cognitive impairments among children. They consume nutrients from children and cause intestinal bleeding, leading to nutrient mal-absorption, nutrient deficiency, cell and tissue destruction, anaemia, intestinal obstruction, abdominal pain, diarrhoea, mental and physical development retardation and other health problems (18). Overall, these factors result in delayed growth, decreased mental development, school absenteeism, and low academic achievement (19).

There are various factors associated with helminthic infection, which includes, lack of safe drinking water, poor environmental sanitation, lack of education and poor socioeconomic status (20–22). Open defecation of human excreta in fields, bushes, forests, ditches, streets, canals rather than use of toilet is the main causes of intestinal parasitic infections (23, 24). Hand washing habits, latrine usage, and contact with soil has been previously acknowledged as additional factors contributing to infection (25). In addition, insufficient health services, as well as lack of the required awareness, due to the absence of effective health education are among the contributing factors for the elevated IPIs among poor communities (20, 22, 26). World Health Organization (WHO) data show, 844 million people (58% living in sub-Saharan Africa) have no access to basic drinking water service while 2.3 billion people still lack access to fundamental sanitary facilities worldwide (27).

In Ethiopia, intestinal helminthiasis remains pressing public health concern. The pooled prevalence of at least one intestinal parasitic infection among school children was 46.1% (22). A study done on intestinal parasites infections (IPIs) of elementary school children in Sidama region showed that about 64.2% of school children were infected by at least one parasite. The prevalence of STHs was 54.7%: *A. lumbricoides* (45%), *T. Trichiura* (25.3%) and *hookworm* (6.1%) were the commonest (21).

In Ethiopia, access to basic sanitary facilities is very limited, particularly in rural areas. According to the 2016 Ethiopian Demography and Health Survey report, only 57% of rural households have access to improved sources of drinking water, and 39% of rural households still lack access to a toilet facility (28). Moreover, about 82% of the Ethiopian population uses unimproved sanitation facilities, and approximately 38.1 million people still practiced open-field defecation (29). These poor sanitation and hygiene conditions may contribute to the high prevalence of intestinal helminth infections in human (30).

To address the high burden of helminthic infection, Ethiopia launched a national school-based deworming program in November 2015. Moreover, efforts have been made to improve water, sanitation and hygiene (WASH). However, despite these ongoing efforts, achieving significant and sustained reductions in prevalence of STHs and other NTDs remains a major challenge both in children and adults in Ethiopia including in Sidama Region where the present study was conducted. Evidence from Sidama region has often limited, and without detailed epidemiological insights from specific districts, national neglected tropical programs may struggle to develop tailored interventions for high-risk population. Furthermore, institution-based data are limited, there is limited evidence on prevalence and risk factors among school children in the study aera. Therefore, this study aimed to determine the prevalence and risk factors associated with intestinal helminthic infections in Tula district, Ethiopia. The findings from this study are expected to fill existing knowledge gaps and enhance context-based planning, resources allocation and targeted intervention strategies.

## 2 Method and materials

### 2.1 Study design

An institution-based cross-sectional study design was employed among schoolchildren at the Primary School in Hawassa, Tula sub city, Sidama Region, Ethiopia. Ethical approval was granted by the Ethical Committee of Yanet-Liyana College of Health Sciences. The reporting of this study follows the criteria described in the Strengthening the Reporting of Observational Studies in Epidemiology (STROBE) (31).

### 2.2 Study area and period

**Figure 1:**
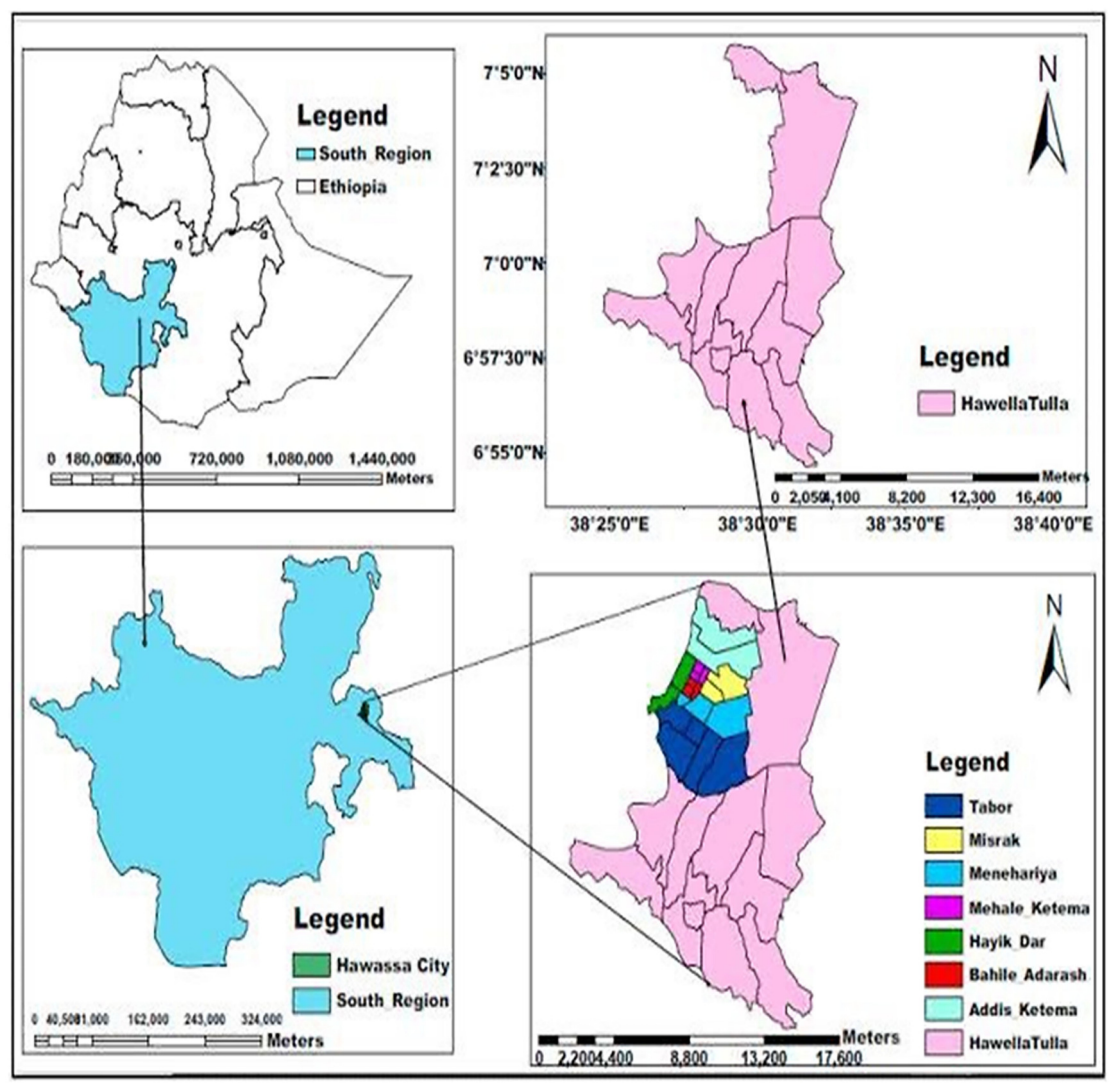
Study area map (54).

The study was conducted from October 1, 2023, to November 30, 2023, G.C., in the Hawela Tula sub city of Hawassa, Sidama Region, southern Ethiopia. It is located 284 km to the south of Addis Ababa and 11 km from Hawassa. Tula has an estimated area of 11,098.1 hectares. It shares borders in the east with Wondo Genet and Malga woredas, in the west with Dore Bafano and Boricha in the south with Shebedino woreda, and in the North with the Tabor subcity of the Hawassa and Oromiya regional states. Administratively, the subcity includes 12 kebeles, of which one kebele is urban and the remaining 11 are rural. According to a report from the subcity administration office, the total estimated population in 2018 was 168,548, of which 87,341 were males and 81,207 were females (54). There is one primary hospital, 12 health centres and 26 public primary schools. For this study, five schools (Tula Junior, Tula Chabicho, Gemeto Aderasha, Gemeto Primary and Bushelo Primary), which represented 20% of the total primary schools found in Tula, were selected (Figure 1).

#### 2.2.1 Populations and study units

All students who were enrolled in public primary schools in the Hawassa, Tula Sub-City, and Sidama regions were the source of the population for this particular study. All public primary school students from grades 1–4 attending their education at the selected schools composed the study population.

All randomly selected primary school students (grades 1–4) were the study units.

##### 2.2.1.1 Inclusion and exclusion criteria

All primary school students (grades 1–4) attending their education in a selected school were included in the study.

All primary school students (grades 1–4) who were deworming or deworming during the last three months were excluded from the study.

##### 2.2.1.2 Sample size determination

In our study, we calculated sample size by using a single population proportion formula. By considered *z* = 1.96 at 95% of CI, *d* = margin of error(5%),non-response rate = 10%, design effect = 2, sample size calculated using 67.7% prevalence from Southern Ethiopia (Eyamo et al., 32) and the final sample size was 740.

The sample size for the second objective was calculated by using Open Epi software using possible determinant factors, which were determined on the same topic, namely, the prevalence and associated factors of intestinal helminthic infections among schoolchildren in different geographical areas of Ethiopia (32–34). We compare the first and second objective sample sizes. We used the first sample size for an adequate and representative sample.

##### 2.2.1.3 Sampling technique

**Figure 2:**
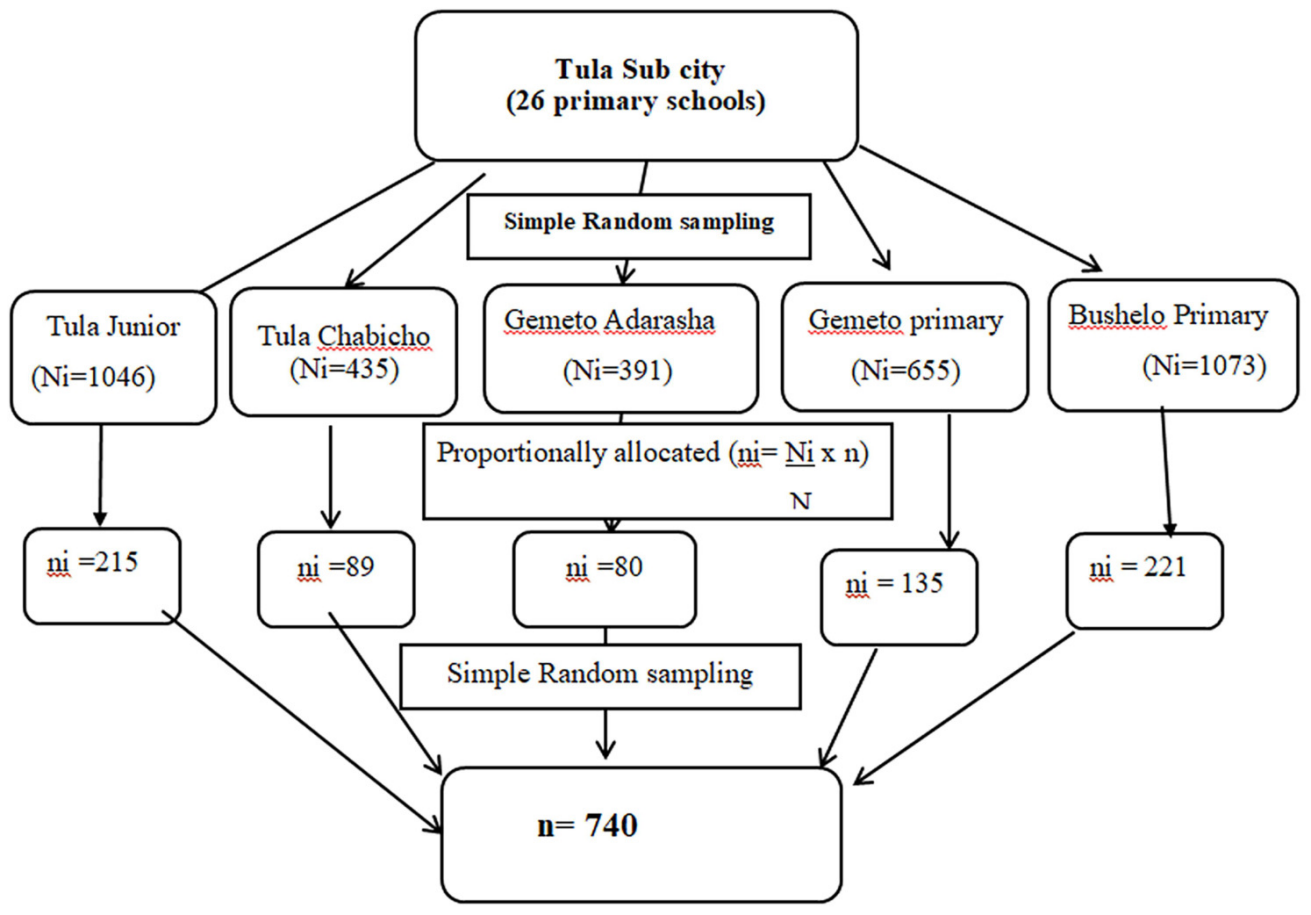
Sampling procedure for the selection of study participants.

A multistage random sampling technique was employed to recruit the study participants. In the first stage, five primary schools, which represented 20% of the total 26 public primary schools found in Tula sub city, were selected by the lottery method and listed as a study population. (Tula Junior, Tula Chabicho, Gemeto Aderasha, Gemeto Primary, and Bushelo Primary School). By using proportionate sampling, the total sample size (*n* = 740) was allocated to each school. Using a simple random sampling technique, class sections to be studied were selected. Finally, the actual number of students needed to participate in the study from each class was selected by using simple random sampling with the class roster as a sampling frame. Students who were absent during the data collection period were replaced by others listed next to them (Figure 2).

### 2.3 Variables

#### 2.3.1 Dependent: intestinal helminthic infections

##### 2.3.1.1 Independent character

**Factors**: sex, age, and residence. The socioeconomic factors included the educational level of the parent, the income of the family, and the family size. **Environmental Factors**:—source of drinking water, water contact frequency, **Personal and Behavioural Factors**: hand washing after latrine, eating raw meat/vegetables, personal and environmental hygiene, fingernail trimming practice, playing with soil, wearing shoes, etc.

### 2.4 Data collection instruments and procedures

The data were collected using an interviewer-administered structured questionnaire, adapted and modified from previously published related literature (35) and from laboratory findings of each stool sample collected and prepared by both wet mount and formol ether concentration methods. The questionnaire included information such as age, sex, grade level of the students, family income, family size and other questions to provide information about their socio-demographic factors and associated risk factors, including behavioural factors and hygiene-related factors, of the study participants regarding intestinal helminthic infection. The data were collected by interviewing each child's parents or guardians via a smartphone application known as the Kobo Tool V.2022, 2.4. During the interview, the children's parents used Sidamigna language. Trained laboratory technologists collected the stool samples.

#### 2.4.1 Stool examination

Following the completion of the questionnaire, every participant in the study received a labelled stool cup along with an applicator stick that was dry, clean, and leak proof. They were then directed to bring around 5 grams of their own recently passed stool. All samples were taken to the Tabor Mother and Child Speciality Centre Hawassa for analysis. A small quantity of every faecal sample was made into wet mounts and analysed to identify helminthic eggs. Two stool samples were used to create two separate smears, each of which was analysed by different technologists. The remaining specimen was stored in a 10% formalin solution for the formol-ether concentration method. The formol-ether concentration technique was utilized for processing every stool sample. During the stool sample collection and laboratory examination procedures, the main researchers were supported by two skilled laboratory technologists and one laboratory technician, all following the standard procedure for sample collection and examination, which also included identifying parasites. One skilled health officer was in charge of supervision.

#### 2.4.2 Direct microscopy

The stool specimen was initially examined macroscopically to assess the presence of worms, consistency, and color. Microscopic examination was then performed to detect and differentiate the stages of certain parasitic organisms. A direct wet mount was prepared by mixing a small amount of stool with a drop of physiological saline (0.85% NaCl solution) on a glass slide, covering it with a coverslip, and examining it under a light microscope at 10× and 40× magnification.

#### 2.4.3 Formal-Ether concentration method

Using an applicator stick, around 3 grammes of pea-sized stool specimens and 10 mL of 10% formalin were combined in a centrifuge tube to emulsify the fresh faeces specimen. Following their emulsification in formalin, the faeces were filtered through gauze into a test tube and spun for two minutes at a relative centrifugal force of around 2,000 rpm. After decanting the supernatant and adding 10 mL of regular saline solution, the sample underwent another centrifugation. Until the supernatant turned clear, the washing process was repeated. A 10% formalin solution (7 mL) was then added, and the mixture was incubated for 5 min. After adding three millilitres of diethyl ether, the tube was sealed with a stopper and given a vigorous one-minute shaking. After removing the stopper, the sample was centrifuged for five minutes at 2,000 rpm. Five minutes were spent letting the tube rest. Four layers were revealed: ether made up the top layer, followed by a plug of detritus in the second, a transparent coating of formalin in the third, and silt in the fourth. A cotton swab was used to remove the debris plug from the test tube's side, and the liquid was extracted, leaving a tiny bit of formalin to suspend the sediment. A pipette was then used to remove the silt. Under a cover slip, a drop of sediment and saline was combined on the slide for analysis. 10× and 40× objectives were used to investigate the helminthic ova. This technique is utilised to verify that a tiny number of eggs are present in the parasites as well as the existence of ova and larvae that are not detectable by direct microscopy (Supplementary Figure 1).

##### 2.4.3.1 Data quality assurance

Five percent of research participants were pretested using appropriately constructed questionnaires in a different location with the identical setup as the Hogoba Primary School in the Tabor sub-city of Hawassa in order to guarantee accurate results prior to the data collecting period. After being produced in English, the questionnaire was translated into Sidamigna, the indigenous language of the research region. The supervisors and data collectors, including laboratory technicians, received two days of training. Standard operating protocols were followed for all laboratory analyses, and laboratory supplies were examined for data that had expired. During data collection, supervision was provided, and a parasitology colour atlas served as a guide (Supplementary Figure 2).

##### 2.4.3.2 Data analysis

After the data collection process ended, the data were exported from the server in excel and SPSS label and imported to SPSS version 27, and the data were cleaned and checked for completeness. Descriptive analysis such as frequency, proportion, mean and SD was calculated. Binary logistic regression was subsequently conducted to assess association between the Intestinal helminthic infections.t and independent variables. In bivariable regression analysis,an indepent variable with *P-value* < 0.25 was entered into the multivariable binary logistic analysis model In multivariable logistic regression, variables with a *P-value* <0.05 were considered significant association with dependent variables. Model fitness was checked by Hosmer and Lemeshow's model of good fit (*p*-value > 0.05). Multi collinearity was checked by linear regression using the variance inflation factor (VIF).

##### 2.4.3.3 Operational definitions

**Prevalence of any intestinal helminthic infections:** is defined as the percentage of individuals within a population who are infected with at least one species of intestinal helminthes (36).

**School-age children** are typically considered to be those between the age range of 5–12 years (36).

**Soil-transmitted helminths:** include four major species of nematodes: *Ascaris lumbricoides*, the whipworm *Trichuris trichiura*, and the hookworms *Necator americanus* and *Ancylostoma duodenale* (37).

## 4 Result

### 4.1 Respondents socio-demographic characteristics

Among the 740 selected study respondents, 726 were included, yielding a response rate of 98.1%. The mean (+SD) age of the study respondents was 8.8 (±2.1) years, with 363 (50.0%) were 5–7 years old. Nearly half (375, 51.7%) were male, and in similar proportion (376, 51.8%) were in grades 3–4. Most study participants (493, 67.9%) resided in urban areas.

Mothers/caretakers educational levels varied, with 154 (21.2%) had no formal education. The majority of mothers/caretakers were housewives (355, 48.9%), while (221, 30.4%) worked as merchants. Similarly, with 128 (17.6%) of fathers' had no formal education. The majority of families comprising fewer than five members, with 493(67.9%) (Table 1).

**Table 1:** Respondents socio-demographic characteristics at primary schools in Hawassa city, Hawela Tula sub city, Sidama region, Ethiopia, 2023.

Variables	Category	Frequency	(%)
Sex of child	Male	375	(51.7)
	Female	351	(48.3)
Child age in years	5–7 years	363	(50.0)
	8–11 years	363	(50.0)
Grades of the students	Grades 1–2	350	(48.2)
	Grades 3–4	376	(51.8)
Residence	Urban	493	(67.9)
	Rural	233	(32.1)
The educational level of the mother/caretaker	No formal education	154	(21.2)
	Elementary	317	(43.7)
	High school	183	(25.2)
	College and above	72	(9.9)
Occupational status of mother/caretaker	Housewife	355	(48.9)
	Merchant	221	(30.4)
	Employed	89	(12.3)
	Farmer	48	(6.6)
	Others	13	(1.8)
Father's educational level	No formal education	128	(17.6)
	Elementary	328	(45.2)
	High school	192	(26.4)
	College and above	78	(10.7)
The occupational status of the father	Farmer	349	(48.1)
	Merchant	227	(31.3)
	Employed	89	(12.3)
	Daily labourer	48	(6.6)
	Others	13	(1.8)

### 4.2 Water, sanitation and hyiene practice

About 64 (8.8%) of the participants were reported as swimming in nearby water bodies. Poor personal hygiene was observed 81 (11.2%), characterized by untrimmed fingernails and uncleanliness, while 37 (5.1%) had visible dirt under their skin. The majority of the study respondents, 517 (71.2%) practice hand washing before meals.

Most respondents (90.1%, 654) used chlorinated tap water, while (9.9%, 72) relied on boiling water. Only, 4.7% (31) had a water source near their latrine. About 192 (26.4%) respondents consumed raw meat and unwashed vegetables. More than half, 398 (54.8%) engaged soil play, though 655 (90.2%) consistently wore shoes, of those, 594 (90.7%) wear closed shoes, 61 (9.3%) wear open shoes, while 61 (9.3%) used open shoes. About 336 (46.3%) of children reported experienced abdominal pain (Supplementary Table S1).

### 4.3 Key Findings on environmental characters

The current study findings indicated that, majority (721, 99.3%) respondents had a latrine. However, ownership patterns highlight disparity, while 546 (75.6%) respondents had private latrine, nearly quarter, 176 (24.4%) respondents reported shared it with neighbours, that may suggesting potential challenges in hygiene management and privacy. In this study waste disposal methods varied significantly: 424 (58.4%) of respondents disposed of refuse waste in an open field, while 57 (7.9%) respondents were used pit, nearly quarter, 178 (24.5%) used a garbage disposal systems, underscoring a need for improved waste management infrastructure. Despite 716 (98.6%) of the respondents had accessing piped water for drinking and cooking, only 31 (4.7%) of them had a water sources near latrine (Table 2).

**Table 2:** Environmental-related factors of schoolchildren at primary schools in Hawela Tula sub—city, Hawassa, Sidama region, Ethiopia, 2023.

Variables	Category	Frequency	(%)
Had latrine	Yes	721	(99.3)
	No	5	(.7)
Ownership of the latrine	Privately owned	546	(75.6)
	Shared with neighbours	176	(24.4)
Dispose of refuse	Pit-hole	57	(7.9)
	Open field	424	(58.4)
	Burning	67	(9.2)
	Garbage	178	(24.5)
Source of water for drinking and cooking	Pipe	716	(98.6)
	Protected well/spring	10	(1.4)
Water (available near latrine)	Yes	31	(4.7)
	No	623	(95.3)

### 4.4 Prevalence of intestinal helminthiasis

**Figure 3:**
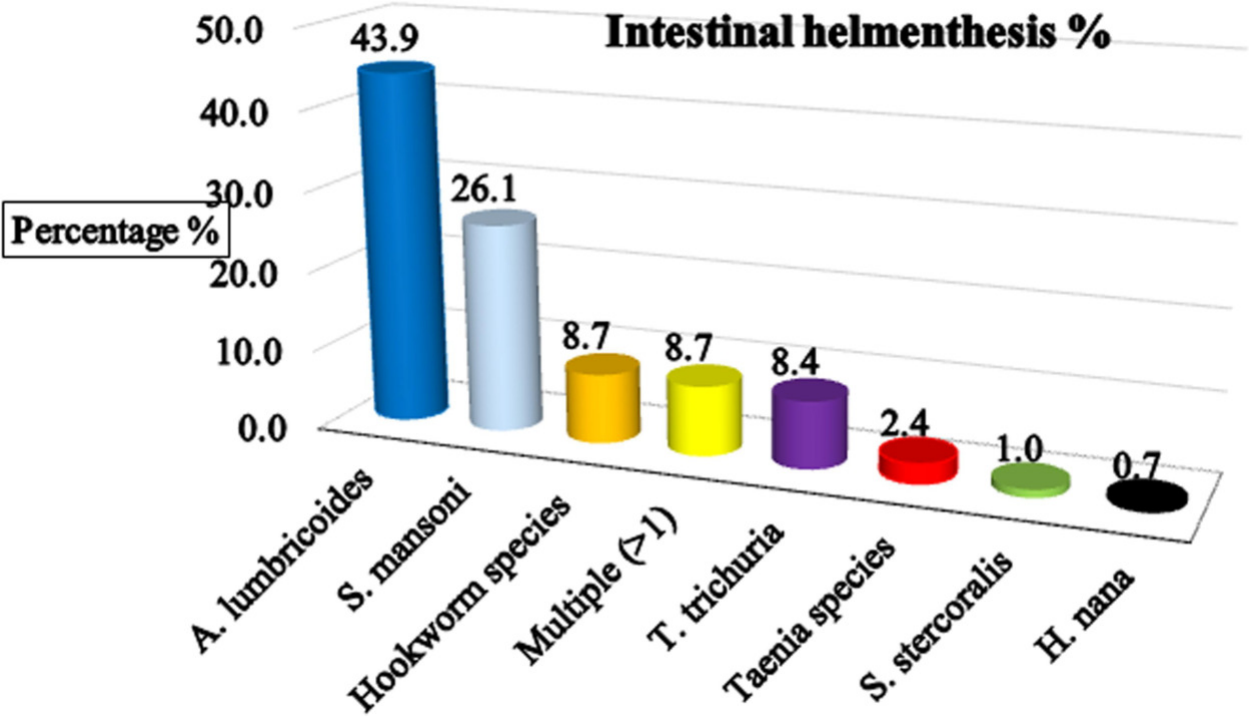
The major identified intestinal helminthic parasite among schoolchildren in Hawassa, Hawela Tula sub-city, Sidama region, Ethiopia, 2023.

The study found a substantial burden of intestinal helminthic infections among schoolchildren, with an overall prevalence of 287 (39.5%) (95% CI: 36.0–43.1). Among the reported prevalence of intestinal helminthic parasite species, 126 (43.9%) of *A. lumbricoides*, which was the leading helminthic pathogen in the area. 75 (26.1%) of the children who were infected with *S. mansoni, 25 (8.7%) of the respondents infected with* hookworm species 25 (8.7%)of the children were infected with more than one species, multiple infections, 24 (8.4%) of the children infected with*T. trichuria*, about 7 (2.4%) of the school children infected with *Teania* species, 1% of the respondnets reported with *S, Stecoralis*, and 0.7% of the children infected with *H.Nana* (Figure 3). This indicates that nearly **two in every five children** were infected, highlighting a significant public health concern in the study area (Supplementary Figure 3).

### 4.5 Factors associated with the prevalence of intestinal helminthiasis

According to the bivariate analysis, the factors that were found to be associated with intestinal helminthiasis and candidates for multivariate analysis were child age in years, student grade, residence status, maternal educational status, family size, untrimmed fingernails and not cleanness, hand washing before the meal, eating raw meat and unwashed vegetables, and child play with soil; these factors had *p* values < 0.25 and were considered candidates for multivariable logistic regression analysis.

After controlling for confounders in the multivariable analysis, lower grades (1–2), maternal educational status, untrimmed fingernails, not washing hands before meals and eating raw meat and unwashed vegetables were significantly associated with intestinal helminthic infection.

The odds of developing intestinal helminthiasis are almost two times greater among lower-grade students than among their counterparts (AOR = 1.53, 95% CI (1.12, 2.09). Children born to mothers with no formal education were approximately 1.5 times more likely to have intestinal helminthiasis [AOR = 1.50, 95% CI (1.05, 2.15)] than were children born to mothers with higher education levels.

Children who had untrimmed fingernails and who were unclean approximately 1.84 times more likely to have intestinal helminthiasis, with AOR = 1.84, 95% CI (1.12, 3.01), and those who did not practice hand washing before meals were also more likely to be exposed to intestinal helminthiasis, with an AOR = 1.90, 95% CI (1.35, 2.68), than were their counterparts. Similarly, those who ate raw meat and unwashed vegetables [AOR = 1.58, 95% CI (1.11, 2.24)] were more likely to have intestinal helminthiasis than their counterparts (Table 3).

**Table 3:** Bivariate and multivariable logistic regression analysis for intestinal helminthiasis among schoolchildren at primary schools in Hawassa city, Hawela Tula sub-city, Sidama region, Ethiopia, 2023.

Variables	Intestinal helminthiasis						
	Yes		No				
	No.	(%)	No.	(%)	COR (95% CI)	AOR (95% CI)	*P* Value
Child age in years							
5–7 years	156	(43.0)	207	(57.0)	1.335 (0.990, 1.799)	1.315 (0.962, 1.798)	0.086
8–11 years	131	(36.1)	232	(63.9)	1	1	
Grades of the students							
Grades 1–2	156	(44.6)	194	(55.4)	1.504 (1.115, 2.028)	1.531 (1.117, 2.099)	0.008*
Grades 3–4	131	(34.8)	245	(65.2)	1	1	
Residence							
Rural	103	(44.2)	130	(55.8)	1.331 (0.970, 1.826)	1.332 (0.954, 1.861)	0.093
Urban	184	(37.3)	309	(62.7)	1	1	
Maternal educational status							
No formal education	71	(44.7)	88	(55.3)	1.577 (1.051, 2.365)	1.502 (1.048, 2.153)	0.027*
Primary education	129	(41.6)	181	(58.4)	1.393 (0.988, 1.963)	1.522 (0.995, 2.330)	0.053
Secondary and above	87	(33.9)	170	(66.1)	1	1	
Family size							
<5	183	(37.1)	310	(62.9)	1	1	
≥5	104	(44.6)	129	(55.4)	1.366 (0.995, 1.874)	1.360 (0.975, 1.897)	0.070
Untrimmed fingernails and not clean							
Yes	46	(56.8)	35	(43.2)	2.203 (1.380, 3.517)	1.837 (1.121, 3.011)	0.016*
No	241	(37.4)	404	(62.6)	1	1	
Children did not practice hand washing before the meal							
No	101	(48.3)	108	(51.7)	1	1	
Yes	186	(36.0)	331	(64.0)	1.664 (1.202, 2.304)	1.904 (1.354, 2.676)	<0.001*
Eat raw meat and unwashed vegetables							
Yes	91	(47.4)	101	(52.6)	1.554 (1.113, 2.169)	1.578 (1.111, 2.241)	0.011*
No	196	(36.7)	338	(63.3)	1	1	
Child play with soil							
Yes	170	(42.7)	228	(57.3)	1.345 (0.995, 1.817)	1.360 (0.991, 1.865)	0.057
No	117	(35.7)	211	(64.3)	1	1	

* Statistically significance at *P*-value less than 0.05.

## 5 Discussion

This research employed an institution-based cross-sectional approach to identify the prevalence and associated factors of Schistosomiasis and Soil-Transmitted Helminthiasis among primary school children in Hawassa, Tula sub-city, Sidama Region, Ethiopia. In this study, a high prevalence of intestinal helminth infections was observed, with *Ascaris lumbricoides* being the most commonly identified parasite. Significantly high infection rates were found among school children in lower grade level (grades 1–2), those whose mothers had no formal education, children with untrimmed or unclean fingernails, who did not wash their hands before meals, or who consumed unwashed vegetables.

The study indicated that the overall prevalence of intestinal helminth infections among school children was 39.5% (95% CI: 36.0–43.1%). This result align with prior studies which reported similar prevalence rates including 39.2% in Nile State, Sudan (38); 45.4% in Gboko, Benue State, Nigeria (39); and 40.5% in the Gurage Zone, southern Ethiopia (40). However, the prevalence observed in the present study was higher than that reported in systematic review and meta-analysis report in Ethiopia (33.35%) (6), as well as studies from Adola town in the Guji Zone, southern Ethiopia (33.91%) (41), Gondar town, Northwest Ethiopia (16.7%) (42), Dembi District, southwestern Ethiopia (30.9%) (43), Jimma Town in southwest Ethiopia (24.3%) (44); and Gedeo Zone, southern Ethiopia (27.6%) (45). Conversely, the prevalence observed in current study was lower than previous studies. For example, the prevalence of intestinal helminthic infections was 84.4% in Mettu town, Southwest Ethiopia (46), 67.7% in Southern, Ethiopia (32) and 56% among schoolchildren in Wonago district, Southern Ethiopia (47). The variation across the studies may be due to difference in sample size, socioeconomic position, hygiene habits, sanitation coverage, and diagnostic techniques employed. Moreover, the discrepancies in infection rates may be influenced by environmental factors such as topography, temperature, humidity, altitude, type of soil, and rainfall, which can affect the transmission dynamic and survival of helminth eggs and larvae.

Children in lower grades (grades 1–2) were more likely to be infected with intestinal helminths compared to those in higher grades (grades 3–4). This finding was consistent with the findings of studies from Port Elizabeth, South Africa (48), Ethiopia (49) and southern Ethiopia (50). This finding may be due to lower awareness of personal hygiene and practice among the children in lower grades. Additionally, children in lower grades may frequently contact contaminated soil during play.

Maternal education significantly associated with intestinal helminths infection. Children born to mothers with no formal education were more likely to have increased intestinal helminthiasis infection than were children born to mothers with higher education levels. This shows that educated mothers are more likely to have better knowledge of hygiene, sanitation, and food handling practices, which in turn protects their children from infection. Similar association have been reported in studies from Ethiopia, where maternal education level was a strong predictor of child parasitic infection (46, 51, 52).

Children with untrimmed or unclean fingernails had higher odds of infection. Untrimmed or unclean fingernails can harbor intestinal parasites eggs and facilitate fecal-oral transmission. Because dirt and debris accumulate under long nails, children may find it more difficult to clean their hands effectively, allowing helminth eggs or larvae from contaminated surfaces to be transferred to the mouth. The findings aligns with studies from southern Ethiopia (50), Jimma town, southwest Ethiopia (44), and Butajira town, south-central Ethiopia (52).

Handwashing practices were another important factor associated with high rates of helminths infection. Children who did not practice handwashing before meals were about two times more likely to be infected. Similar findings have been reported in studies conducted in the Gedio Zone, southern Ethiopia (45), Adola town, Ethiopia (41), and in rural Debre Tabor, Northwest Ethiopia (53). This finding indicates that the critical role of proper hand hygiene in preventing fecal-oral transmission of intestinal helminths.

Eating and food preparation habits also played a significant role in risk of infection. Children who consumed unwashed vegetables or raw meat were more likely to have intestinal helminthiasis. The finding is consistent with studies conducted in southern Ethiopia (47, 51) and southwest Ethiopia (46). The finding linked to the consumption of contamination food items with helminth eggs or larvae. Raw or undercooked or improperly handled meat, can harbor different stages of parasites. Unwashed vegetables can harbour soil or faecal matter that may contain helminth eggs.

In general, our study found high prevalence of intestinal helminths, underscore the importance of integrating school-based interventions. The intervention may focus on health education that increases parental awareness specially targeting mothers with lower education, and promotion of hygiene practices such as regular handwashing and nail trimming. Moreover, the health education programs also should work on safe food handling before consumptions, particularly for vegetables and meat products. Additionally, deworming campaign should prioritize young children in lower grades.

## 6 The strength and limitations of the study

The use of multistage random sampling, standardized questionnaires, and rigorous laboratory analyses was performed by laboratory technologists, and high-calibre investigation tools were used. In addition, data collection is conducted by using a digital Kobo collection tool that enhances data quality. However, the cross-sectional nature of the study limits the ability to establish causality or determine the temporal relationship between variables. Data were collected only during the dry season (October–November); helminth transmission is often higher in rainy periods, so the prevalence might be underestimated. There may be a potential recall and social desirability bias during the interview, particularly regarding the age of the child and the socioeconomic status of the participants. Moreover, data collected between October and November (dry season) may underestimate peak transmission during rains. The current study uses microscopy-based detection (wet mount and formol-ether concentration) lacks sensitivity for light infections compared to Kato-Katz or molecular (PCR) methods; this may underestimate prevalence, Only grades 1–4 from five schools were included; results may not generalize to older children.The study was conducted at an institutional level; therefore, the findings may not generalizable.

Self-reported hygiene and dietary behaviors (e.g., hand washing, eating raw vegetables) were provided by parents or guardians, introducing information bias. Some potential confounding factors (e.g., nutritional status, prior deworming history beyond three months, household crowding) were not controlled for. Prevalence was reported, but infection intensity (egg count per gram of feces) was not measured, limiting the epidemiological depth.

## 7 Conclusion

This study found a high prevalence of intestinal helminth infection among primary school children in southern Ethiopia, with *Ascaris lumbricoides* being the predominant parasite. Lower grade level, lack of maternal education, untrimmed or unclean fingernails, poor handwashing habits, and consumption of unwashed vegetables were factors associated with intestinal helminth infection. These findings underscore the need for incorporating school-based interventions focusing on hygiene education, safe food handling, and regular deworming programs and teaching curriculum. Integrated efforts to improve sanitation and access to clean water are also essential to reduce the burden of intestinal helminthiasis.

## Data Availability

The original contributions presented in the study are included in the article/Supplementary Material, further inquiries can be directed to the corresponding author.
