# Supplementary material for: Schistosomiasis and soil-transmitted helminthiasis prevalence and associated factors among school children in the Hawela Tula sub-city, Ethiopia: a cross-sectional study
Source: Front Epidemiol. 2025 Nov 25;5:1514964. doi: 10.3389/fepid.2025.1514964 (PMC12685865; doi:10.3389/fepid.2025.1514964)
Supplement: Supplementary file 1 [file Datasheet1.docx]

**Supplementary Figure 1: Picture of Formol ether concentration**


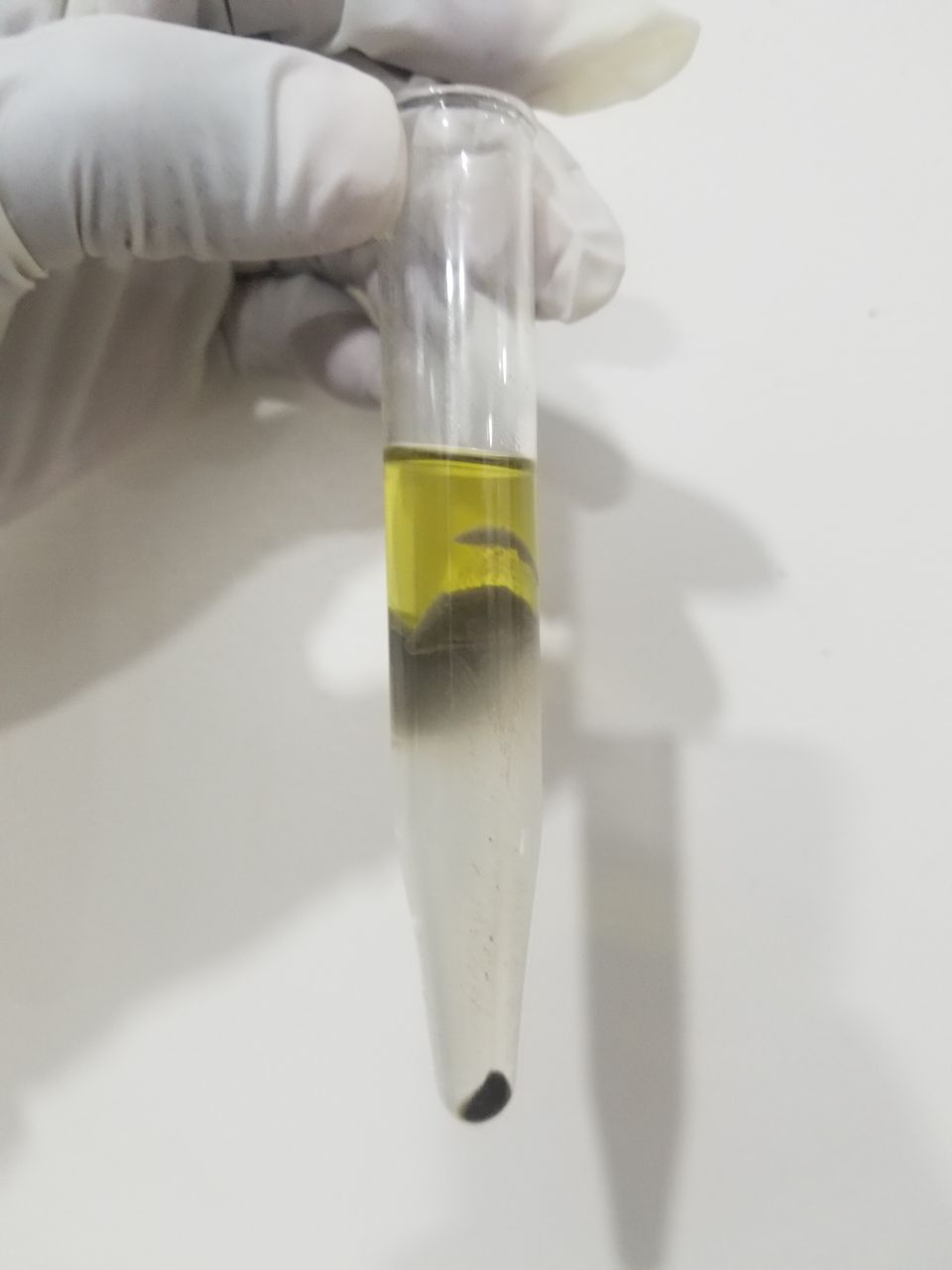


Sediments

Formalin Solution

Fecal Debris

Ether solution

**Supplementary Figure2: Color Atlas Of Parasitology**


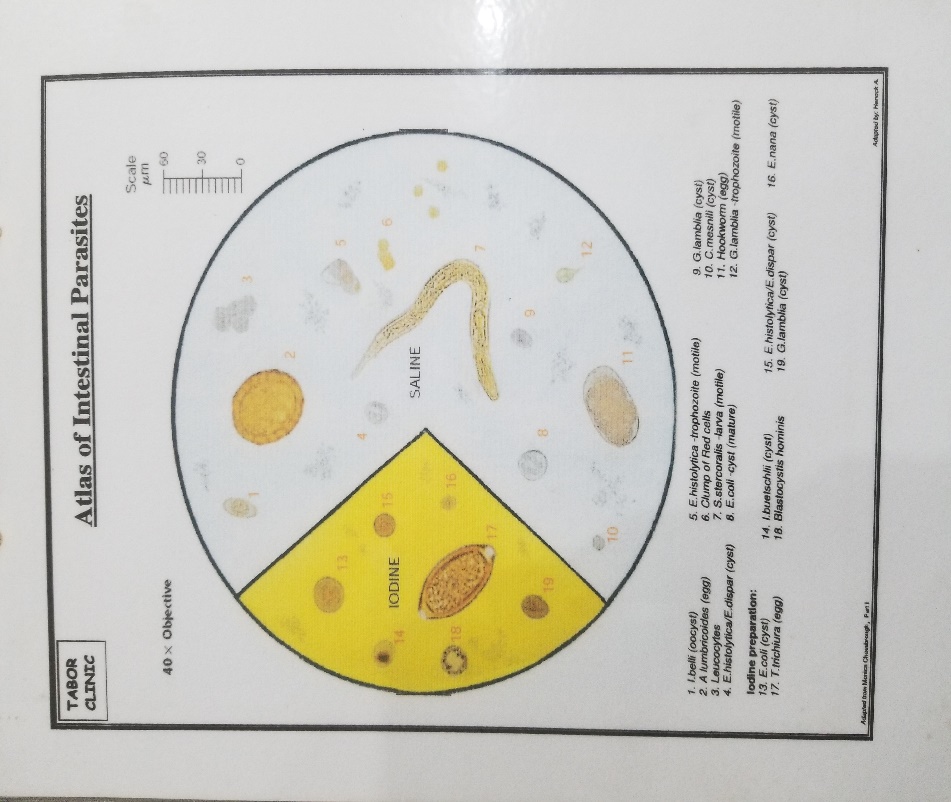

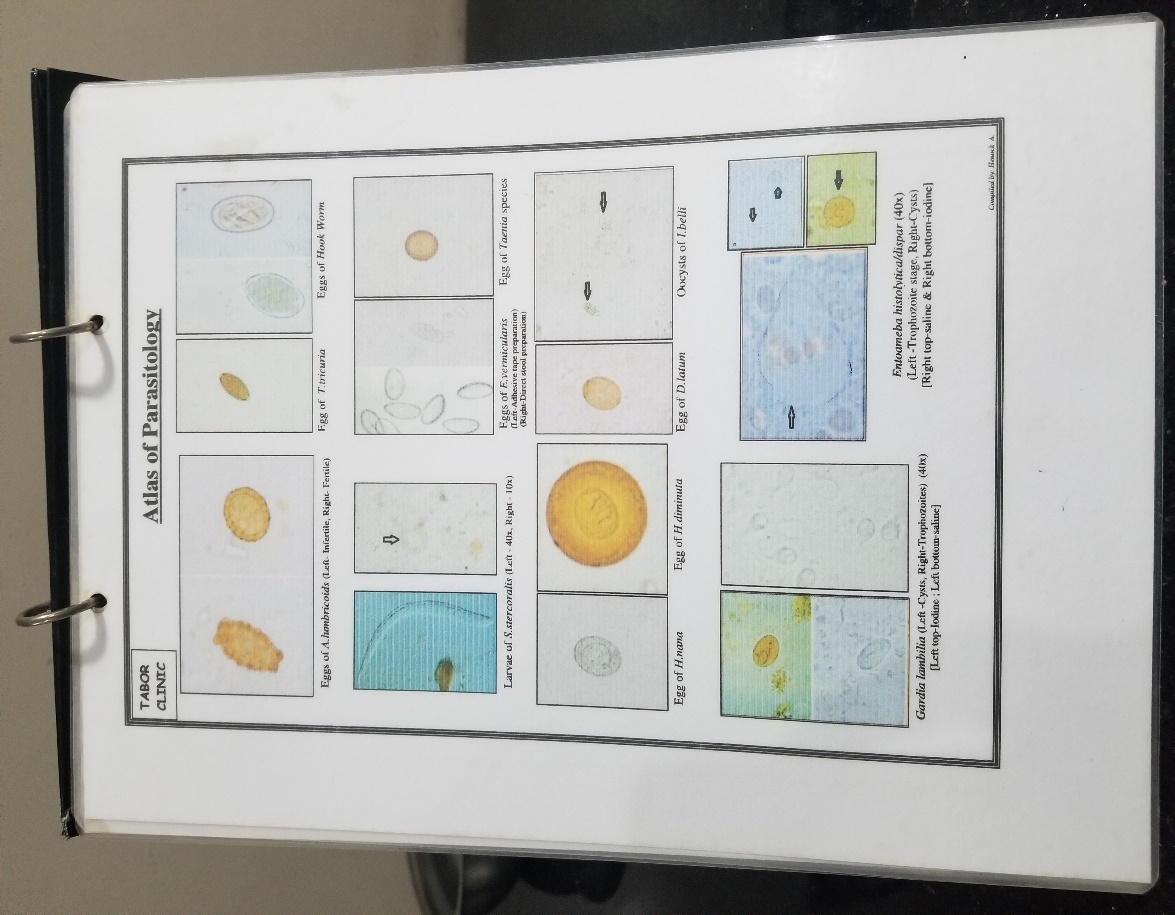


**Supplementary Figure 3**: Helminthes parasite Detected by Microscop, among schoolchildren in Hawassa, Tula sub-city, Sidama region, Ethiopia, 2023.


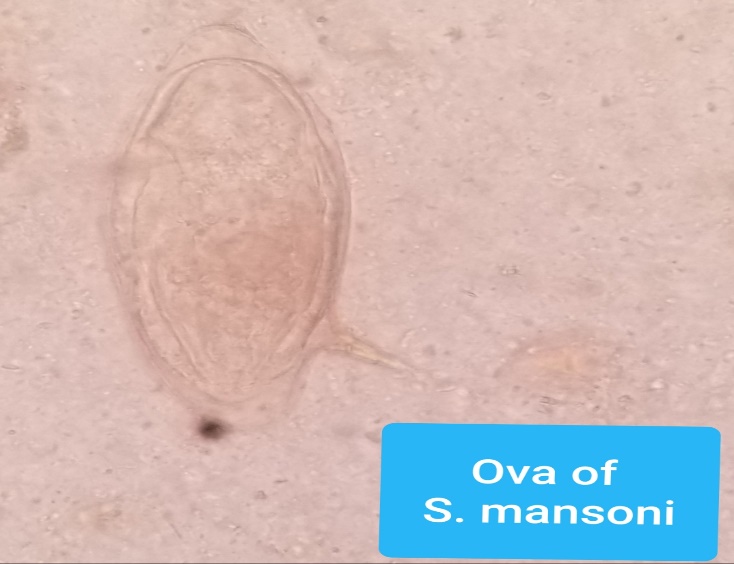

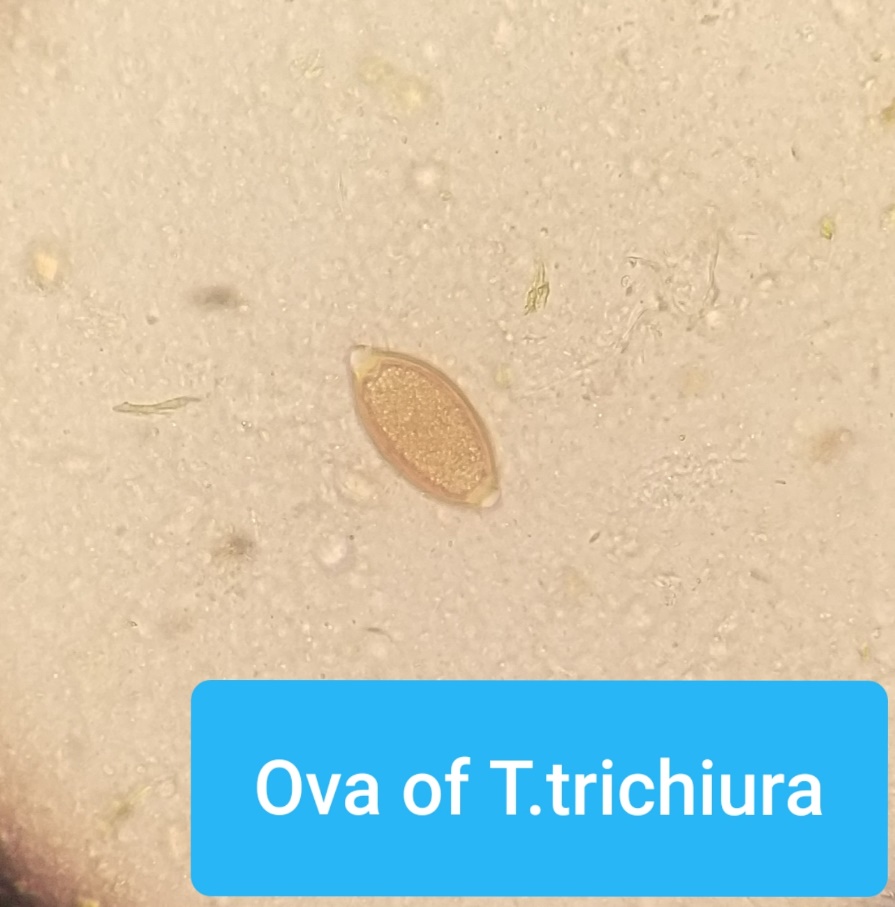

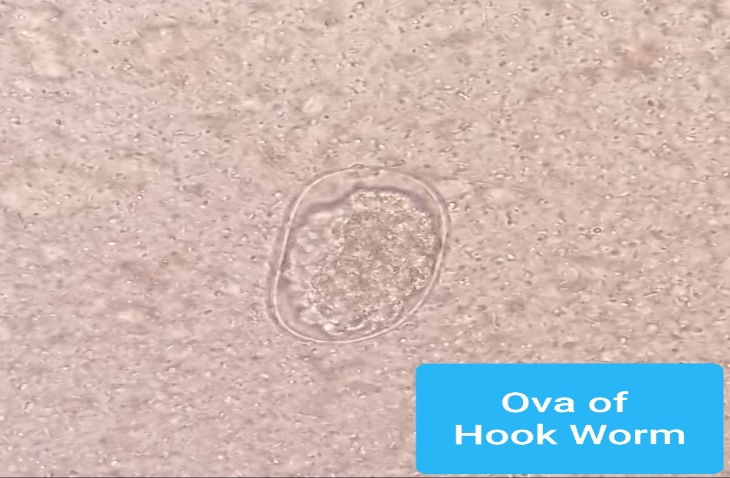

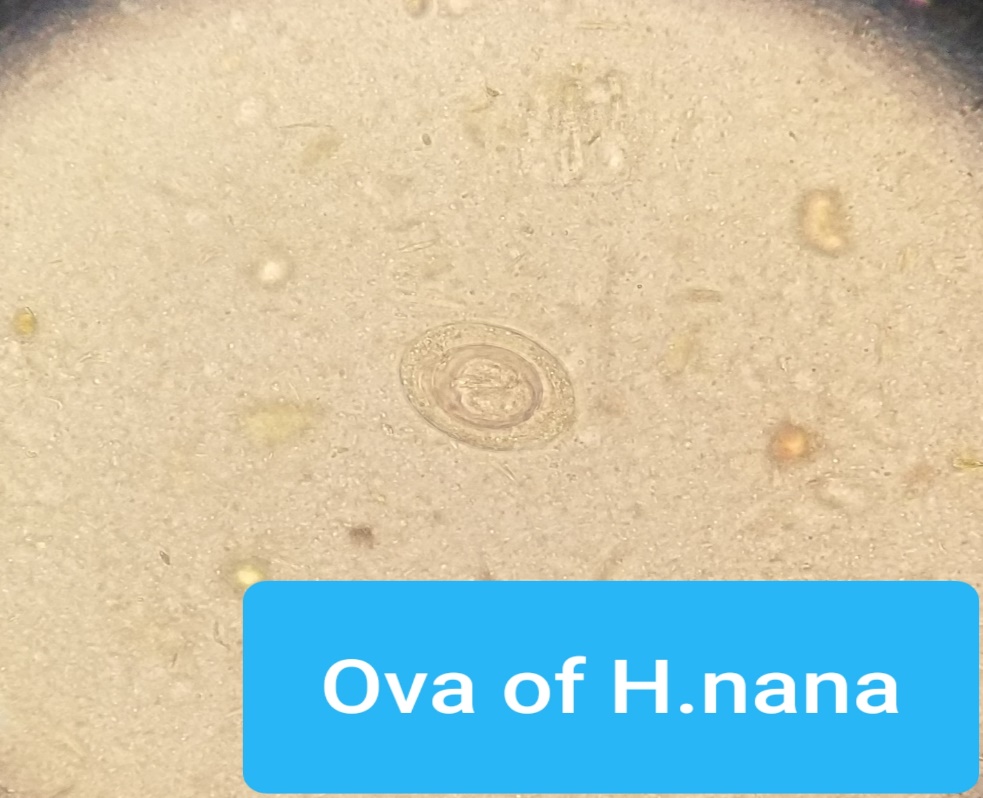


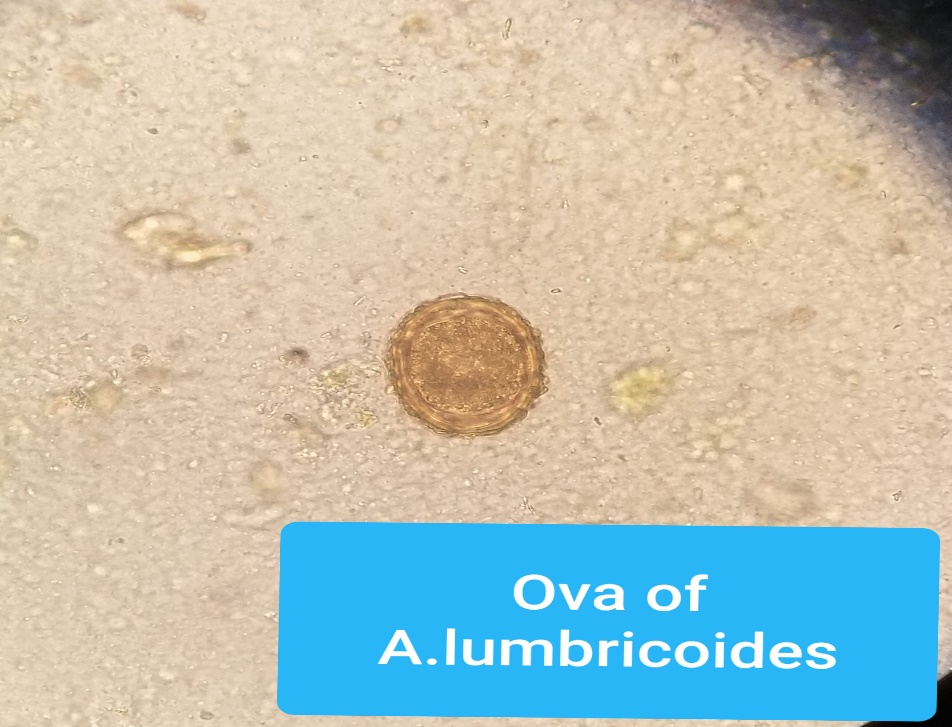

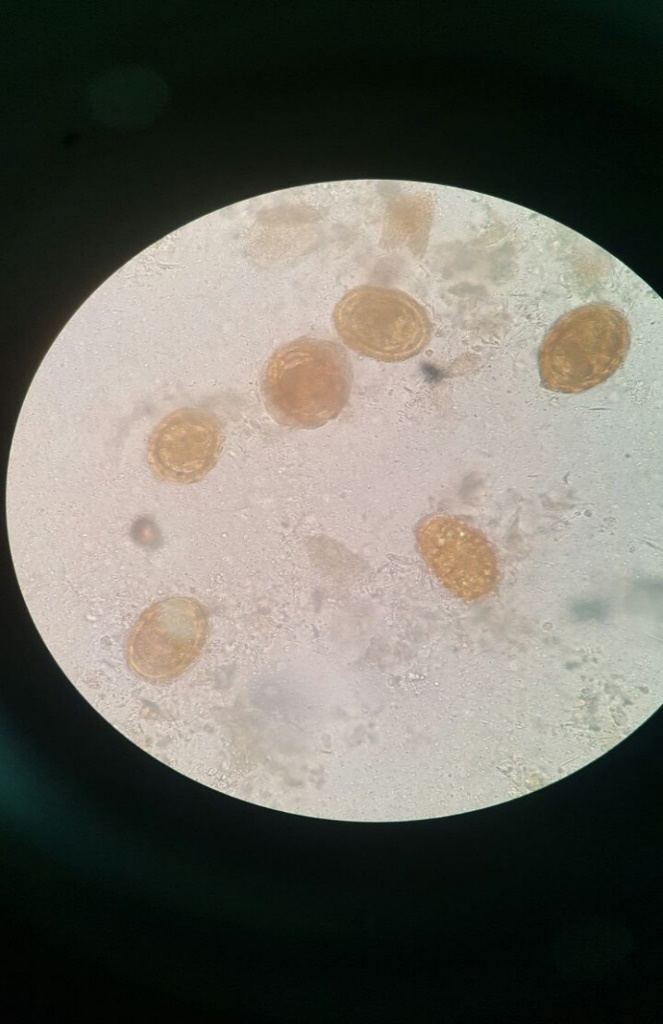


**Ova of A. lumbricoides after 10% formol ether concentration method done**
