# Supplementary material for: Schistosomiasis and soil-transmitted helminthiasis prevalence and associated factors among school children in the Hawela Tula sub-city, Ethiopia: a cross-sectional study
Source: Front Epidemiol. 2025 Nov 25;5:1514964. doi: 10.3389/fepid.2025.1514964 (PMC12685865; doi:10.3389/fepid.2025.1514964)
Supplement: Supplementary file 2 [file Table1.docx]

**Supplementary Table 1** : Individual- and behavioural-related factors for schoolchildren at primary school in Hawassa city, Tula sub city, Sidama region, Ethiopia, 2023

| **Variables** | **Category** | **Frequency** | **(%)** |
| --- | --- | --- | --- |
| Swim in nearby water bodies | Yes | 64 | (8.8) |
|  | No | 662 | (91.2) |
| Untrimmed fingernails and not clean | No | 645 | (88.8) |
|  | Yes | 81 | (11.2) |
| Dirt matter under your skin | Yes | 37 | (5.1) |
|  | No | 689 | (94.9) |
| Children practice hand washing before the meal | No | 209 | (28.8) |
|  | Yes | 517 | (71.2) |
| Treat drinking water | Chlorinated tap water | 654 | (90.1) |
|  | by boiling | 72 | (9.9) |
| Water source available near the latrine | Yes | 31 | (4.7) |
|  | No | 623 | (95.3) |
| Eat raw meat and unwashed vegetables | No | 534 | (73.6) |
|  | Yes | 192 | (26.4) |
| Child play with soil | No | 328 | (45.2) |
|  | Yes | 398 | (54.8) |
| Children always wear shoes | Yes | 655 | (90.2) |
|  | No | 71 | (9.8) |
| Type of shoes | Open shoe | 61 | (9.3) |
|  | Closed shoe | 594 | (90.7) |
| Had any abdominal pain | Yes | 336 | (46.3) |
|  | No | 390 | (53.7) |
|  |  |  |  |
